# The combined treatment with ketogenic diet and metformin slows tumor growth in two mouse models of triple negative breast cancer

**DOI:** 10.1186/s41231-024-00178-8

**Published:** 2024-06-22

**Authors:** Karen Schmidt, Amber Thatcher, Albert Grobe, Pamela Broussard, Linda Hicks, Haiwei Gu, Lesley G Ellies, Dorothy D. Sears, Leonid Kalachev, Eugene Kroll

**Affiliations:** 1 Division of Biological Sciences, University of Montana, Missoula, MT, USA; 2 Silverlake Research Corporation, Missoula, MT, USA; 3 College of Humanities and Sciences, University of Montana, Missoula, MT, USA; 4 College of Health Solutions, Arizona State University, Phoenix, AZ, USA; 5 Department of Pathology, University of California San Diego, San Diego, CA, USA; 6 Department of Mathematical Sciences, University of Montana, Missoula, MT, USA; 7 Present address: Okinawa Institute of Science and Technology Graduate University, Okinawa, Japan

**Keywords:** Hypoxic tumor, Glycolytic tumor, Systemic glucose limitation, Ketogenic diet, Metformin

## Abstract

**Background:**

Many tumors contain hypoxic microenvironments caused by inefficient tumor vascularization. Hypoxic tumors have been shown to resist conventional cancer therapies. Hypoxic cancer cells rely on glucose to meet their energetic and anabolic needs to fuel uncontrolled proliferation and metastasis. This glucose dependency is linked to a metabolic shift in response to hypoxic conditions.

**Methods:**

To leverage the glucose dependency of hypoxic tumor cells, we assessed the effects of a mild reduction in systemic glucose by controlling both dietary carbohydrates with a ketogenic diet and endogenous glucose production by using metformin on two mouse models of triple-negative breast cancer (TNBC).

**Results:**

Here, we showed that animals with TNBC treated with the combination regimen of ketogenic diet and metformin (a) had their tumor burden lowered by two-thirds, (b) displayed 38% slower tumor growth, and (c) showed 36% longer latency, compared to the animals treated with a ketogenic diet or metformin alone. As a result, lowering systemic glucose by this combined dietary and pharmacologic approach improved overall survival in our mouse TNBC models by 31 days, approximately equivalent to 3 years of life extension in human terms.

**Conclusion:**

This preclinical study demonstrates that reducing systemic glucose by combining a ketogenic diet and metformin significantly inhibits tumor proliferation and increases overall survival. Our findings suggest a possible treatment for a broad range of hypoxic and glycolytic tumor types that can augment existing treatment options to improve patient outcomes.

## Introduction

Aggressively growing tumors develop hypoxic microenvironments due to insufficient and haphazard tumor vascularization [1–3]. Chronic tumor hypoxia promotes metastasis [4, 5], increases angiogenesis [6, 7], inhibits the immune response [8, 9] and interferes with apoptosis [10]. Furthermore, tumor-derived micrometastases are initially avascular and, therefore, exist in a state of acute hypoxia [11–13]. The hypoxic status of a tumor also correlates with resistance to chemo-, radio- and immunotherapies, advanced stages of malignancy and poor clinical prognosis [10, 14, 15].

Cancer cells readily adapt to hypoxic conditions via activation of hypoxia-inducible factors [16–18]. Downstream signaling promotes overexpression of hexose transporters [19, 20] and the eventual depolarization of mitochondrial inner membranes, which inhibits oxidative phosphorylation (OXPHOS) [21]. This effect, first noted by Louis Pasteur [22], forces hypoxic cells to rely on oxygen-independent glycolysis for their energetic and anabolic needs [13, 23, 24]. Some cancer cell types evolve to retain the glycolytic phenotype even in the presence of oxygen, as shown by Otto Warburg [25].

Provided enough glucose is available, hypoxic tumor cells rapidly produce ATP despite the inefficiency of glycolysis compared to OXPHOS [26]. Additionally, the increased glycolytic flux provides ample feedstocks for cellular components [24]. This reliance of hypoxic tumor cells on high glucose flux is *a metabolic vulnerability* and offers new strategies for cancer therapy.

Taking advantage of the relative inefficiency of glycolysis, we postulate that a reduction in systemic glucose may check the growth of hypoxic tumors and their metastases while sparing normal tissue. Properly vascularized and oxygenated tissues can catabolize other nutrients such as fatty acids, ketone bodies, glutamine and lactate, all of which require OXPHOS to produce ATP [27]. Certain tissues, such as the brain, predominantly use glycolysis but are able to switch to ketone bodies upon glucose shortage [28, 29] or survive in a mildly hypoglycemic environment [30]. This is supported by the fact that mild hypoglycemia (>60 mg/dL) is well-tolerated in mice (See Results) and is not considered life-threatening in humans [31].

To control systemic glucose, all possible sources of carbohydrates must be addressed. Exogenous (dietary) sources can be controlled with low-carbohydrate (ketogenic) diets, and endogenous glucose production can be partially inhibited by metformin, an antidiabetic agent. Clinically relevant doses of metformin reduce endogenous glucose output by suppressing gluconeogenesis via mitochondrial glycerophosphate dehydrogenase (mGPD) with a resultant change in the redox state of the cytoplasm [32] and indirectly activating starvation signaling [33]. Individually, ketogenic diets and metformin are well-tolerated in humans [34], but their anticancer properties, used separately, have been relatively marginal [35–40].

To test whether lowering systemic glucose could affect hypoxic tumors, we applied the combination regimen of a ketogenic diet and metformin to two mouse models of triple-negative breast cancer (TNBC). TNBC often metastasizes, and is ultimately responsible for more than 90% of breast cancer deaths [41]. As TNBC is genetically heterogeneous, effective therapies are lacking [42]. TNBC breast tumors are also frequently hypoxic and glycolytic [2], making this type of breast cancer a suitable model to study the effects of reducing systemic glucose.

In this work, we describe the effect of inducing mild, controlled hypoglycemia in vivo in two TNBC mouse models by analyzing tumor latency, tumor growth rate and overall survival. Then, we verify the direct glucose dependency of hypoxic breast cancer cells on abnormally high glucose concentrations in vitro.

## Methods

### Animals

The use of experimental animals followed guidelines in the National Institutes of Health *Guide for the Care and Use of Laboratory Animals*. The experimental protocol was approved by the University of Montana Institutional Animal Care and Use Committee, and work was conducted in an AAALAC-certified facility. Forty 4 weeks-old female FVB mice were used for injection experiments (2 tumors per animal), and twenty 4 to 6 weeks-old female PyMT transgenic mice (B6.FVB/N-Tg(MMTV-PyMT)634Mul/LellJ) that randomly produce mammary tumors were used in this study (Jackson Laboratories, Bangor, ME). We used a lower number of PyMT animals because this model produces, on average, 4 tumors in one animal.

### Tumor cell injection

FVB mice were anesthetized with 5% isoflurane until recumbent and unresponsive to a toe pinch. Anesthetized animals were placed in a supine position and injected with 0.5 ×10^6^ Met-1 cells in 2 mg/mL Matrigel (total volume = 50 μL) into L4 and R4 mammary pads using a 25-gauge needle.

### Tumor oxygenation levels

An OxyLite monitor (Optronix, Oxford, UK) was used to measure tumor tissue oxygenation by detecting molecular oxygen in tissues based upon quenching of light emitted by a fluorescent dye, where the quenching is proportional to the pO_2_ and temperature of the surrounding tissue. Animals were anesthetized with 5% isoflurane in oxygen and maintained at 1–2% isoflurane throughout the procedure. Once animals were unresponsive to a toe pinch, a 22-gauge angiocath was inserted into the tumor lengthwise and the needle was removed. The probe was then inserted into the angiocath to the desired position, and the angiocath was removed while holding the probe in place. The probe was maintained in the desired position for 3 min for the reading to stabilize, the reading recorded, and the probe retracted an additional 3 mm. This procedure was repeated to obtain three or four measurements in tumor tissue (depending on tumor size). Similar measurements of nearby subcutaneous tissues were taken as controls. Ambient air was also measured and recorded for comparison.

### Diet and metformin dosing

FVB and PyMT transgenic mice were randomized into four groups: 1) C group – control group maintained on a standard mouse chow diet (Teklad 2020x), 2) M group – standard chow plus metformin, 3) K group - Ketogenic diet (Teklad TD.96355) and 4) KM group - Ketogenic diet plus metformin. PyMT animals were apportioned to groups so that their ages were equally distributed among all groups. Diet and water were available *ad libitum*. Animals in the M and KM groups were given metformin in drinking water at 5 g/L supplemented with 2 g/L Stevia for palatability. Water consumption was measured every two days, and the concentration of metformin was adjusted accordingly.

### Metformin level in mouse blood

Plasma samples were mixed with methanol and centrifuged. Supernatants were vacuum-dried and reconstituted in 40% PBS/60% acetonitrile. The quality control (QC) sample was pooled from all available samples. External calibration solutions were used to determine the absolute concentrations of metformin. LC-MS/MS was performed on an Agilent 1290 UPLC-6495 QQQ-MS (Santa Clara, CA) system in hydrophilic interaction chromatography (HILIC) mode on a Waters XBridge BEH Amide column. The mobile phase was composed of Solvents A (10 mM ammonium acetate, 10 mM ammonium hydroxide in 95% H_2_O/5% acetonitrile) and B (10 mM ammonium acetate, 10 mM ammonium hydroxide in 95% acetonitrile/5% H_2_O), and the auto-sampler temperature was kept at 4°C The mass spectrometer was equipped with an electrospray ionization source. Targeted data acquisition was performed in multiple-reaction-monitoring mode. The whole LC-MS/MS system was controlled by Agilent Masshunter Workstation software (Santa Clara, CA). The extracted MRM peaks were integrated using Agilent MassHunter Quantitative Data Analysis.

### Vital signs and tumor volume measurements

Mouse activity was observed daily and scored according to the Murine Behavior Ethogram, with blood glucose and body weight recorded at least weekly for each mouse. Blood glucose was measured before feeding the animals (i.e., fasting glucose). Tumor size was best represented by volume, which we selected as the indicator for tumor burden. To calculate volumes, two orthogonal diameters were measured with calipers with an estimated precision of 6.4% (See Supplemental Materials). Each tumor was evaluated by palpation in the third dimension (height) as flat, ovoid or round. Depending on the shape of the tumor, one of the following formulae were used to calculate volume: “Flat” $\frac{\pi •x•\left(\frac{y^{2}}{4}\right)}{6}$, “Ovoid” $\frac{\pi •x•y^{2}}{6}$, or “Round” $\frac{\pi •x•y•\left(\frac{x+y}{2}\right)}{6}$, where *x* is the largest diameter and *y* is the smallest.

### Modeling tumor growth

An exponential tumor growth model [43] was fit to the data for all treatment groups (C, M, K and KM - see Diet and metformin dosing) with the assumption that tumors proliferate at a constant rate for a particular treatment group, while estimated tumor burden (the volumes of all tumors on a mouse) was specific to each mouse. For transgenic animals, to account for randomness in tumor initiation, the time of tumor initiation was adjusted to “0” when the tumor burden (the cumulative volume of all tumors in one animal) was 10mm^3^ (the initial tumor burden). The following exponential model was used:

$$x_{gi}\left(t_{j}\right)=x_{gi}\left(T\right)•\text{exp}\left(k_{g}•\left(t_{j}-T\right)\right),$$

where $x_{gi}\left(t_{j}\right)$ was the tumor burden of the *i*-th mouse from each treatment group (*g* = C, M, K and KM) measured at a time point $t_{j}$. These tumor burdens were estimated during model fitting. Parameter $k_{g}$ (1/day) is the tumor growth rate constant for each group. MATLAB nlinfit.m (v. R2018a) was used to fit model equations to data to estimate growth rate constants $k_{g}$ and the initial tumor burden for each animal $x_{gi}\left(T\right)$. Standard errors for estimated parameters and statistically reliable inferences about tumor growth rates were obtained using the Delta method [44] under the assumption of normality.

#### Tissue culture.

MET-1 cells (mouse MMTV-PyMT breast cancer cell line [45]) were seeded in 8 T-25 flasks at 30–50 % confluence in complete DMEM (4.5% glucose, 10% FBS) and allowed to reach confluence with one medium change. The medium was then replaced with complete DMEM containing either 0, 0.5, 1.0 or 4.5g/L glucose in duplicate. One set of four flasks (hypoxic) was placed at 37°C in sealed containers with a Gaspak EZ (Beckton Dickinson) to absorb oxygen and an anaerobic indicator strip to confirm the lack of oxygen. The duplicate set of flasks (aerobic) was incubated in the presence of oxygen in standard conditions. To monitor cell death, we chose to use the physiological method of cell attachment to the surface, as the vital stain Trypan Blue is not ideal for measuring cell viability under 80% [46]. Cell death was monitored as follows: after 19-hour exposure to culture conditions as described above, the culture medium with floating cells was pulled out of the flask, centrifuged, and the cell pellet was transferred to a fresh flask in standard conditions (DMEM, 4.5% glucose, aerobic) and monitored for cell attachment daily for one week. Furthermore, the initial flask received standard DMEM medium with 4.5% glucose and was aerobically incubated for 1 week to observe any growth of the cells still attached to the surface, if any.

## Results

### MET-1 mouse breast cancer tumors are hypoxic

**Fig. 1:**
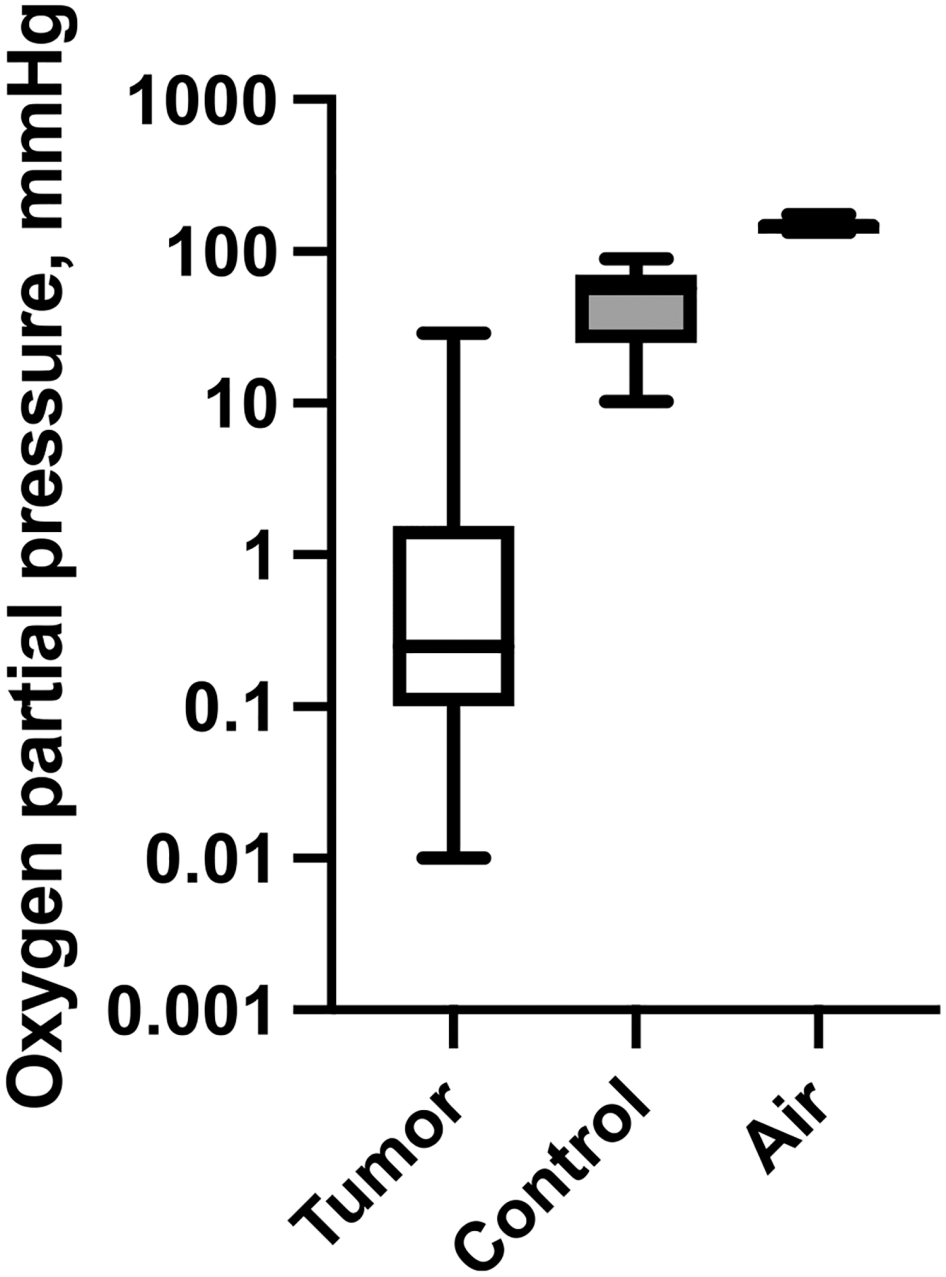
PyMT orthotopic injection tumors display a very low median oxygenation level compared to control (muscle tissue in the vicinity of the tumor). Boxplots depict partial oxygen pressure in respective tissues. The middle line is the median, boxes span the interquartile range, whiskers show the full range of values. To allow for better visualization of the tumor oxygenation range of tumors, the Y axis is logarithmic

To assess the oxygenation state of tumors in our mouse models, we measured the oxygen partial pressure (pO_2_) in six developed breast tumors after orthotopic injection of the PyMT breast cancer cell line MET-1 [45] in FVB mice and compared it to normal tissue. The median partial oxygen pressure in the tumor tissues (pO_2_) was 0.25 mmHg (*n*=40, Interquartile range (IQR) 0.10–1.25), while the median pO_2_ for subcutaneous tissue (control) was 57.0 mmHg (*n*=13, IQR=25.4–65.8). The pO_2_ of the surrounding air was 155 mmHg (*n*=11, IQR=139–156) (Fig. 1). While several tumor tissue measurements were as high as in normal tissue, the median pO_2_ was significantly lower (*p*<0.0001, Mann-Whitney). Consistent with previous studies [1, 47], these data show that the median tumor tissue oxygenation level in our breast cancer mouse model is approximately one order of magnitude lower than in normal tissue.

### A ketogenic diet-metformin combination regimen delays tumor development.

Based on the causal relationship between hypoxia and glucose dependency and the hypoxic nature of our mouse model tumors, we predicted that reducing available glucose would inhibit the growth of hypoxic tumors. To test this, we compared the tumor growth effects of combined ketogenic diet plus metformin treatment (KM) with ketogenic diet alone (K), metformin treatment alone (M), or control (C) in two in vivo mouse models of triple-negative breast cancer.

Animals receiving metformin displayed serum metformin concentrations comparable to previous determinations [48], ranging from 14.8 to 21.8 μM, which approximates human metformin serum concentration at a clinically relevant 1.5g/70kg b.w. dose [49].

**Fig. 2:**
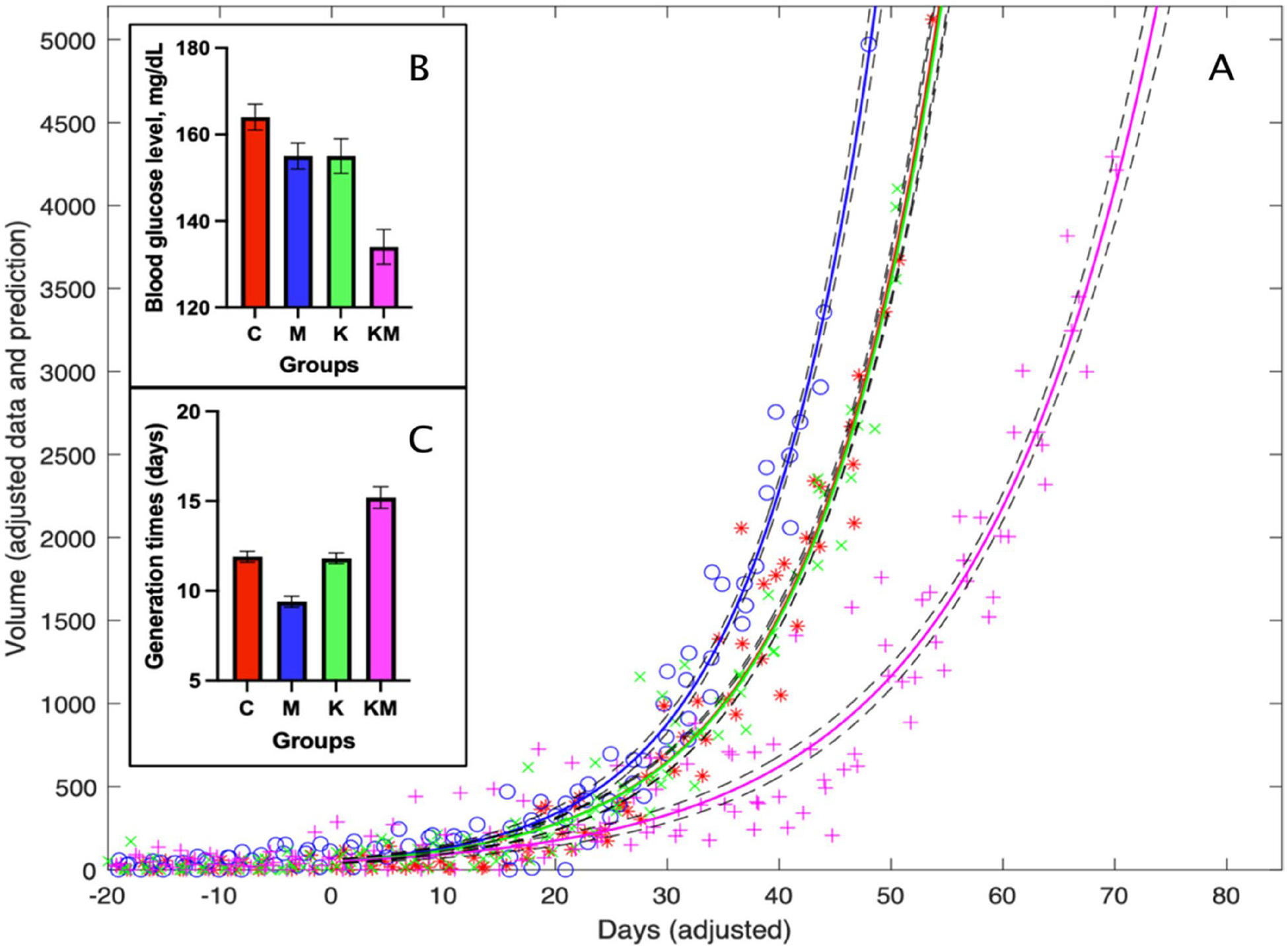
Tumor burden increases at a slower rate in the ketogenic diet/metformin group than in other groups. Red – control (C), blue – metformin only (M), green – ketogenic diet only (K), pink – ketogenic diet plus metformin (KM). **A** Time series model fitted curves depict cumulative tumor volumes for groups C (*), M (o), K (x) and KM (+) (mm^3^). Due to the inherent randomness of tumor initiation in this mouse model, we have assigned day “0” for each animal to be equal to a cumulative tumor volume of 10 mm^3^ (See Methods). This makes apparent the difference in the growth rate constant values. Dashed lines indicate 95% confidence bands. **B** Differences in blood glucose levels between groups. **C** Differences in generation times between groups

Mean blood glucose (BG) levels decreased significantly only in the combination ketogenic diet and metformin (KM) group. For FVB animals, the average BG level in the KM group was 123±6 mg/dL, vs. the average for all other groups at 148±3 mg/dL. For PyMT transgenic animals, the average BG level in the KM group was 117±6 mg/dL *vs*. the average for all other groups at 150±11 mg/dL (Fig. 2B). The lowest BG value in the KM group reached 67.2 mg/dL without an apparent change in animal behavior, as scored using the Murine Behavior Ethogram.

We first estimated tumor burden and growth rates in female PyMT transgenic mice that develop random, human-like, hyperplastic mammary adenocarcinomas with lung metastases within the first three months of life [50]. The total tumor burden (sum of tumor volumes per animal) was not significant between the control (C), metformin-only (M) and ketogenic diet-only (K) groups. In contrast, the mean tumor burden in the ketogenic diet plus metformin group (KM) was 33.4±3.4% of the mean tumor burden in all other groups throughout the experiment (30 measurements). This is a conservative estimate because animals from control groups with large tumors or large overall tumor burden were euthanized earlier, artificially decreasing the tumor burden ratio. To address this and to make firm statistical inferences, we assessed tumor accumulation using an exponential growth model (See Methods).

Due to the inherent randomness of tumor initiation in this mouse model, we have assigned day “0” for each animal to be equal to a cumulative tumor volume of 10 mm^3^ (See Methods). Suppl. Fig. 1 shows growth curves without adjusting for the time of tumor initiation. Then we fit model parameters to the data and estimated tumor generation times (the inverse of growth rate constants): C group, 11.9±0.3 days; M group, 9.4±0.3 days; K group, 11.8±0.3 days and KM group, 15.2±0.6 days. Pairwise differences in tumor generation times for the KM group vs. any other group were significantly different (*p*-values <10^−7^ [z-test]). The combined ketogenic diet plus metformin regimen significantly delayed tumor development compared to other groups (Fig. 2A and C).

Upon the experiment termination, we isolated mouse lungs and analyzed them for the appearance of metastases. The results are provided in Supplementary materials (Suppl. Fig. 2).

### Survival is extended on the ketogenic diet-metformin regimen.

**Fig. 3:**
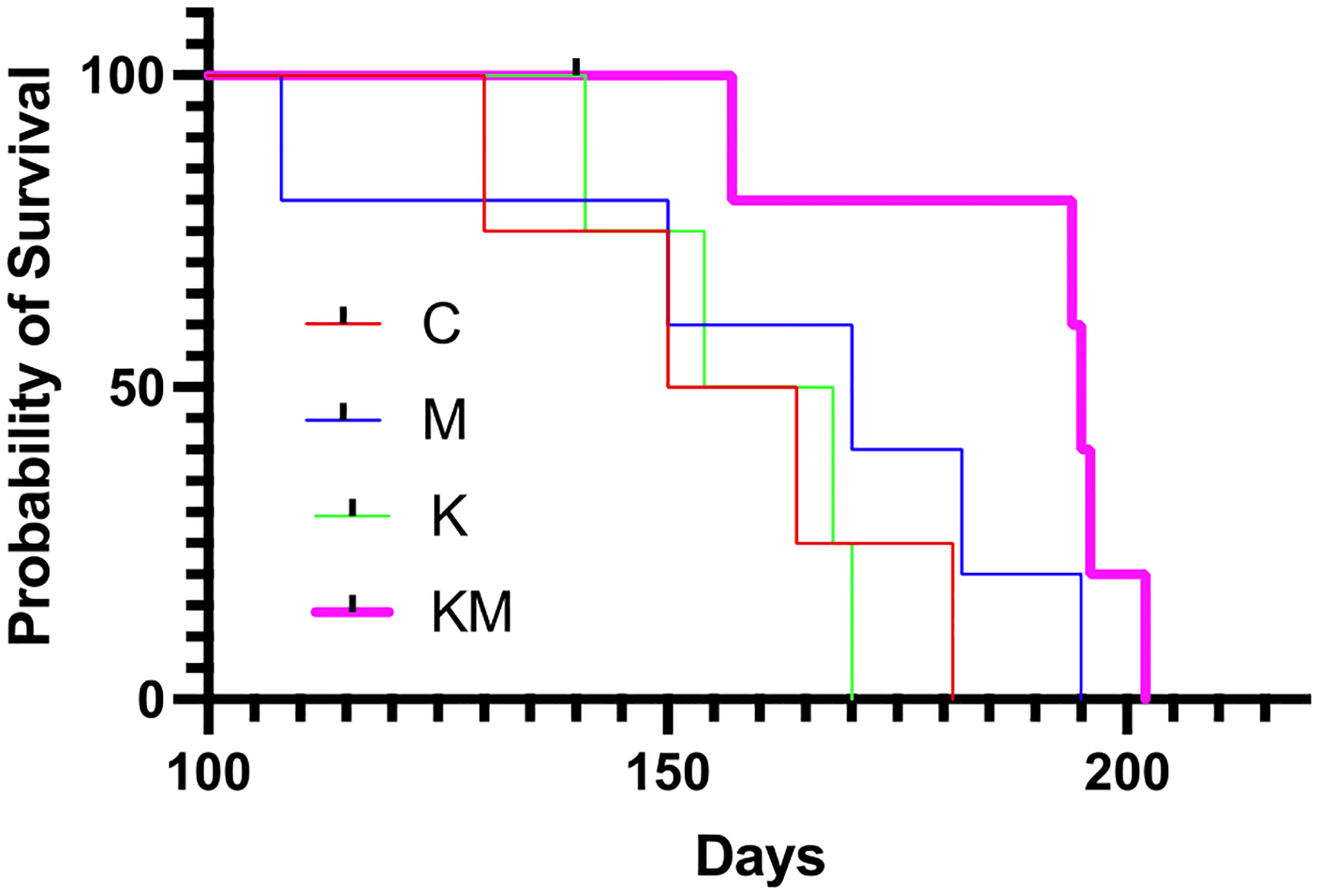
Age-matched animals on ketogenic diet and metformin survive longer than animals in other groups. Time (in days) was adjusted by birth date. Red – control (C), blue – metformin only (M), green – ketogenic diet only (K), pink – ketogenic diet plus metformin (KM)

Second, we estimated overall survival in female PyMT transgenic mice. Median survival time for each animal from its birthdate to the time it had developed a cumulative tumor mass of 20% of its body weight were: C group - 157 days, M group - 170 days, K group - 161 days and KM group −195 days. The difference in survival times between KM and the other groups was statistically significant (*p*-value of 6.89×10^−5^ *χ*^2^ = 15.84, log-tank test) (Fig. 3).

### Tumor latency is also extended on the ketogenic diet-metformin regimen in an orthotopic injection model

Third, we estimated tumor latency, i.e., the period during which the tumor remains undetected, operationally defined here as the number of days for individual tumors to reach a detectable volume of 100 mm^3^. Tumor latency is an important parameter in clinical applications related to cancer prevention efforts. For this experiment, we used the orthotopic injection model because injected tumors have a more uniform initiation and growth pattern than the random PyMT model we used in previous experiments. To synchronize the onset of tumors, we orthotopically injected MET-1 breast cancer cells (bearing the same PyMT construct in their genome as the PyMT transgenic animals) into the L4 and R4 mammary glands of naive FVB mice (2 tumors per mouse). Once tumors became detectable, we recorded their dimensions, converted them to volumes, fit the exponential model parameters to these data (see Methods) and then estimated the time it took cumulative tumor volumes for each animal to reach the detectable level of 100 mm^3^.

**Fig. 4:**
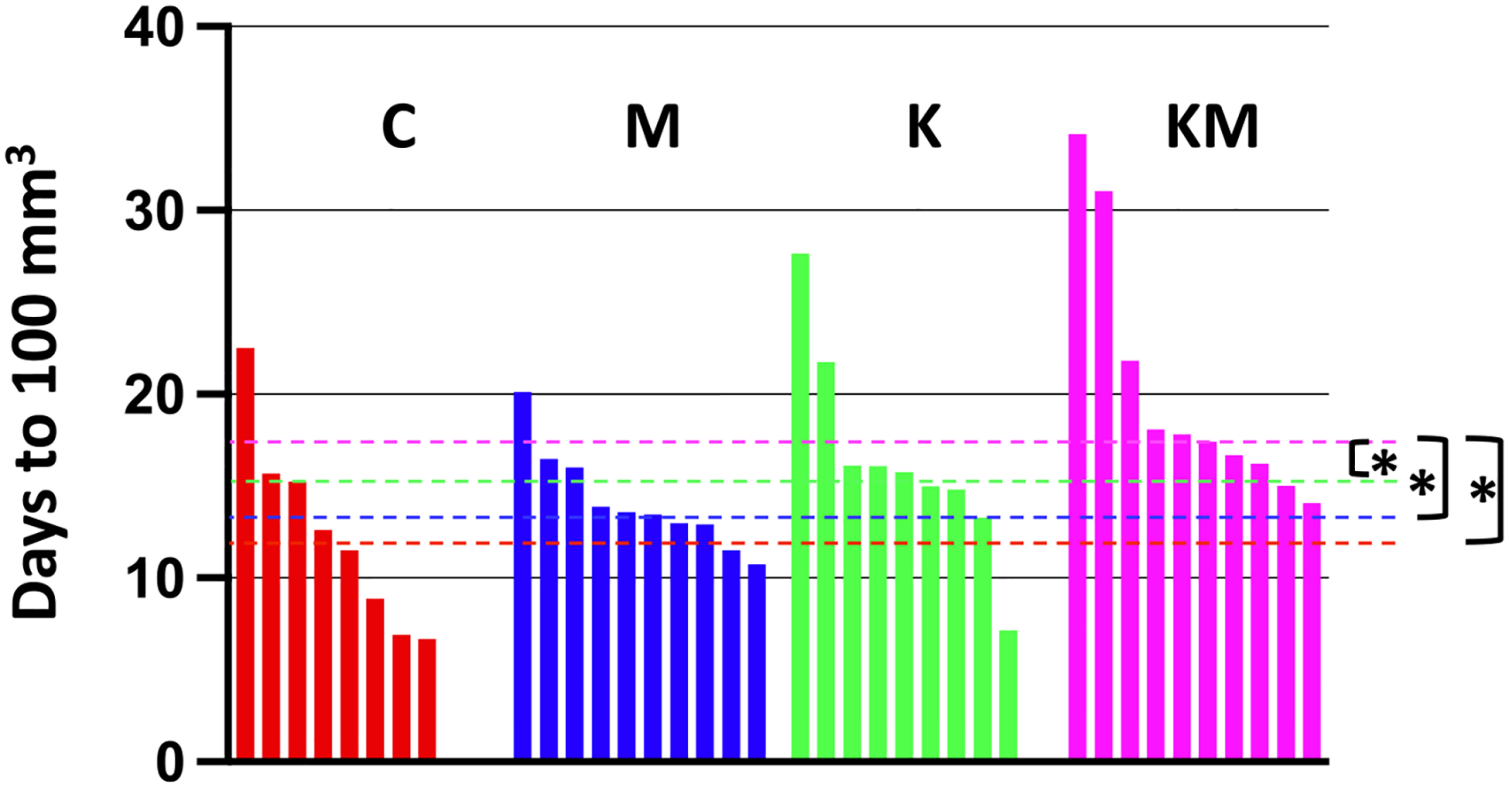
A Tumor latency in the orthotopic injection model. Tumor latency (time to reach a detectable 100 mm^3^ total tumor volume) is longer in the KM group compared to the other groups. Each vertical line represents the data from a separate mouse. Three out of forty animals were dropped out of this experiment because at least one of their two tumors failed to grow. Red – control (C), blue – metformin only (M), green – ketogenic diet only (K), pink – ketogenic diet plus metformin (KM). Dashed lines represent median values for each group. Asterisks denote statistically significant differences at 0.05 significance level (*p*-values are in the text)

The median tumor latency was significantly longer for the KM group animals than other groups (KM vs. C, *p*=0.006; KM vs. M, *p*=0.002; KM vs. K, *p*=0.04, one-tailed Wilcoxon rank sum test). These data confirm that the ketogenic diet plus metformin group exhibited a significantly prolonged latency in tumor growth compared to other groups (Fig. 4).

### Hypoxic, but not normoxic, cancer cells in culture depend on abnormally high glucose to survive.

To confirm that the observed growth inhibition of hypoxic tumors stems from lower available glucose rather than lower insulin [51, 52] or a modulation of the immune response mediated by the glucose consumption-dependent N-glycosylation [53], we replicated the oxygen-starved tumor microenvironment in vitro. Ordinarily, conventional tissue culture conditions offer a hyperoxygenated and hyperglycemic environment, which is far from what tumor cells may experience *in situ*. Cell lines are traditionally grown at a much higher oxygen partial pressure, ~150 mmHg in the atmosphere vs. ~50 mmHg in normal tissue and can be much lower in tumor tissue [54]. Moreover, most culture media contain 4.5 g/L glucose vs. ~1 g/L glucose in the blood and even less in cancer tissues [1].

To model *in situ* tissue microenvironments, we incubated the same MET-1 mouse breast cancer cell line that was used in the injection experiments in a hypoxic chamber with different concentrations of glucose in the DMEM medium (0, 0.5, 1.0 and, for the control, the conventional 4.5 g/L), either in the normal (aerobic flasks) or a low (hypoxic flasks) oxygen atmosphere. The epithelial MET-1 cell line requires cell attachment for viability (*pers. comm*). After 19 h, aerobic flasks with all glucose concentrations showed no indications of cell detachment at all glucose concentrations, as evidenced by medium color and 100% cell adherence. In contrast, hypoxic flasks with glucose concentrations of 0, 0.5 and 1.0 g/L displayed a deep pink media color with cells detached from the flask. However, the hypoxic flask with 4.5 g/L glucose appeared yellow, indicating partial acidification, with no detached cells. To test the viability of detached cells in all flasks, we attempted to rescue the cells by transferring them to a fresh medium with 4.5% glucose and aerobically incubating them for an additional 8 hours and then microscopically observing if cells reattached to the surface. In hypoxic flasks with glucose concentrations of 0, 0.5 and 1.0 g/L, detached cells failed to reattach or grow, indicating that they were non-viable. Complete DMEM with 4.5% glucose was also added to the original flasks to rescue any cells that may still be attached. In the original flasks that contained hypoxic cells with glucose concentrations of 0, 0.5 and 1.0 g/L, no cell attachment or growth was detected with added complete DMEM after one week, in contrast to the hypoxic flasks with 4.5 g/L glucose and all aerobic flasks. These results show that, under hypoxic conditions, MET-1 cells require abnormally high glucose concentrations to survive and that lowering glucose levels in hypoxic conditions leads to cell death.

## Discussion

Aggressive tumor proliferation leads to insufficient tumor vascularization, resulting in chronic tumor hypoxia, which initiates a metabolic shift in cancer cells to become highly glycolytic. Here, we showed that lowering systemic glucose by the simultaneous reduction in dietary carbohydrates and inhibiting gluconeogenesis significantly delays the development of hypoxic breast cancer in vitro and in vivo and may, potentially, inhibit the growth of metastatic nodes in the lungs.

The results of this study demonstrate that hypoxic tumor tissues are susceptible to even mild glucose limitation. Using two aggressive breast cancer mouse models, we showed that a glucose-lowering regimen consisting of a *combination* of two modalities -- a low carbohydrate (ketogenic) diet and metformin -- decreased tumor burden by 2/3 compared to the control or each modality alone. Moreover, tumors in the combination ketogenic diet-metformin group grew 38% more slowly, resulting in an additional 31 days of the median overall survival. This life extension equates to more than three human-equivalent years [55], a significant increase over the current median TNBC survival of 18 months [42]. Additionally, we showed that the median latency of breast tumors in mice using our combination treatment increased by 36% compared to the median latency of other groups. Then, we confirmed that breast cancer cells rely on an abnormally high glucose level to survive in a hypoxic environment in tissue culture. Lastly, since micrometastases are hypoxic due to the lack of vascularization, we obtained preliminary evidence that lung metastasis may also be delayed (see Supplementary materials).

Limiting glucose with a combination of a ketogenic diet plus metformin regimen to slow cancer growth has been independently proposed [56, 57], and this combination regimen has been safely used in humans for a different purpose [58]. Furthermore, timed metformin dosing during transient hypoglycemia caused by intermittent fasting strongly inhibited the melanoma-derived tumors [59]. Other ways to limit systemic glucose levels are also under investigation. Several studies described the direct cytotoxic action of metformin in low glucose conditions in different models, supporting our findings in breast cancer models [60, 61]. Additionally, glycolytic tumors have been targeted by inhibiting glycolysis [62], the PI3 Kinase/Akt/mTORc growth signaling pathway [51], or by blocking glucose transport [63, 64]. However, as with conventional chemotherapies, tumor evolution can circumvent these targeted approaches, leading to cancer recurrence. Additionally, these molecular approaches may be ineffective or toxic, as some molecular targets are redundant or indiscriminate, and some normal cell types may also rely on these activities. In contrast, lowering systemic glucose via the combined regimen proposed here adopts an “organismic” view of cancer [65] by safely modifying organismal physiology rather than targeting a unique cancer activity.

Confirming our findings, diabetic cancer patients taking metformin exhibit a significantly lower incidence of hepatic, colorectal, mammary and pancreatic cancers and increased survival from colorectal, pulmonary and prostate cancers than those on other antidiabetic medications that do not inhibit gluconeogenesis [66, 67]. The most probable explanation is that diabetic patients tend to control their carbohydrate intake better than the general population [68], boosting the metformin anticancer effect. It follows that a low carbohydrate ketogenic diet in combination with metformin may potentiate the metformin anti-carcinogenic action in cancer patients regardless of their diabetic status, as we observed in our mouse models.

An alternative explanation is that a decrease in insulin levels caused by low glucose slows tumor growth. This would mean that in the presence of insulin, the normoglycemic and hypoxic environment should allow cancer cells to proliferate. However, our in vitro experiments show that the normoglycemic (1g/L) insulin–containing DMEM growth medium did not support hypoxic PyMT cancer cells. Instead, to survive, MET-1 breast cancer cells required a “diabetic” 4.5g/L glucose level in these conditions to survive. This observation implies a direct effect of glucose levels on cancer cell growth rather than the indirect effect of lower insulin. While insulin is important in the promotional stage of breast tumorigenesis, a large proportion of advanced ER-negative breast adenocarcinomas do not show a mitogenic response upon insulin signaling in culture [69]. Moreover, hyperinsulinemia tends to be irrelevant to breast cancer risk for premenopausal women while potentially increasing it for post-menopausal women [70]. Evidence in cell culture, mice and humans demonstrates that hyperglycemia is a *bona fide* cancer risk factor, at least for ER-negative breast cancer such as TNBC.

Another alternative explanation is that lower glucose availability may modulate protein glycosylation patterns, which affect a multitude of processes from cell attachment to cell signaling, metabolism and the immune response to cancer cells [53], thus affecting the dynamics of tumor growth. With our in vitro experiments, we ruled out that the immune response to a change in N-glycosylation patterns due to lower glucose availability is involved in slower tumor dynamics, at least in MET-1-derived tumor models. However, other effects of protein glycosylation, such as glucose import by glycosylated symporters, may still provide a plausible explanation.

While we observed a significant decrease in tumor burden, growth rate and an increase in tumor latency with a mild decrease in systemic glucose using a combination of a clinically relevant dose of metformin and a ketogenic diet, the treatment did not inhibit tumor growth altogether. One explanation is that properly oxygenated and, therefore, nonglycolytic tumor cells would not be susceptible to this regimen. Since well-oxygenated, proliferating cancer cells can be targeted by chemo-, radio- and immunotherapies, our metabolic regimen is a natural candidate for combination with these therapies for synergistic therapeutic effects. Additionally, since tumor micrometastases are also hypoxic [5, 18, 21], lowering systemic glucose may affect tumor metastasis similarly to affecting the primary tumor (see Supplementary Materials), increasing the regimen’s potential. Finally, this metabolic regimen may be similarly effective against a broad range of other FDG-PET-positive (glycolytic) tumors in other organs [10, 18, 71].

## Supplementary Material

Supplementary material

## Data Availability

All data and materials will be made available provided agreements are in place.
