# Supplementary material for: The combined treatment with ketogenic diet and metformin slows tumor growth in two mouse models of triple negative breast cancer
Source: Transl Med Commun. Author manuscript; Available in PMC 2024 Nov 21. (PMC11580796; doi:10.1186/s41231-024-00178-8)
Supplement: Supplementary material [file NIHMS2032994-supplement-Supplementary_material.docx]

**
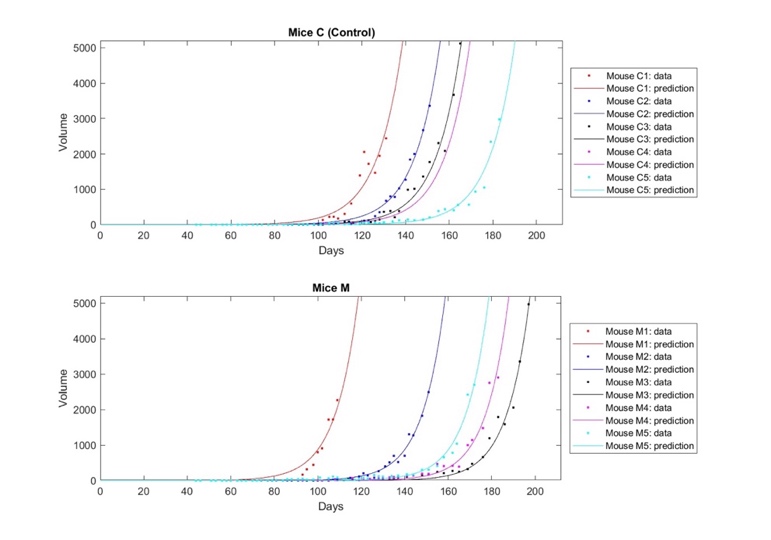

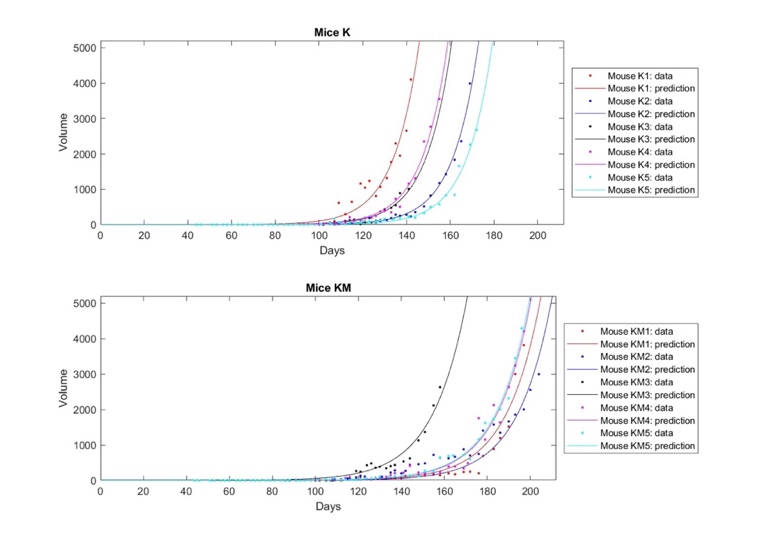
Results of exponential tumor growth model fitting to experimental data for individual PyMT transgenic mice.**

**Supplementary Fig. 1.** Model predictions of tumor growth for individual PyMT transgenic mice in all treatment groups. All time points were age-adjusted such that the time series data for each animal were shifted according to the difference in birthdates.

**
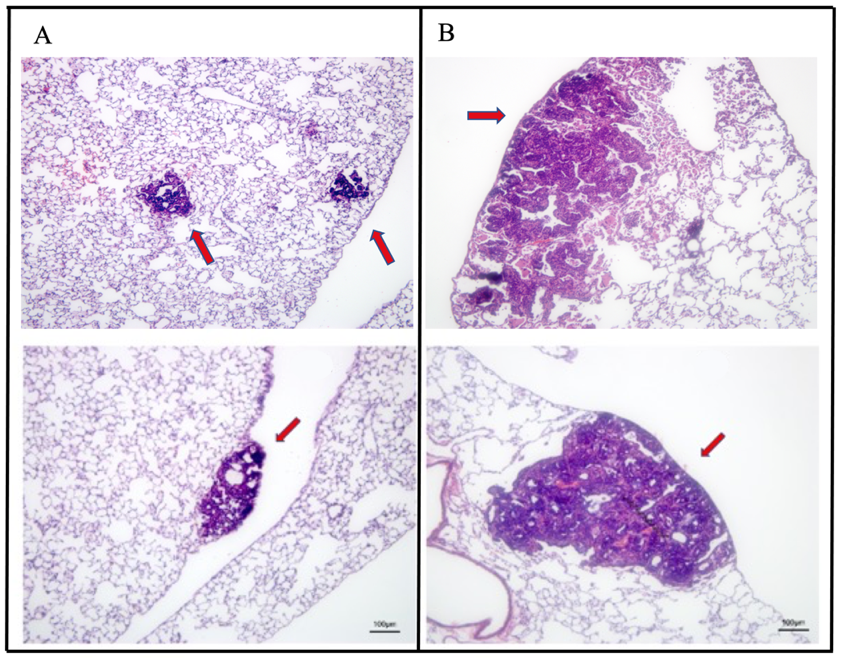
Histological determination of lung metastases***.*

**Supplementary Fig. 2.** Lung metastases are smaller in the lungs of mice treated with the glucose-lowering regimen than in the control. A. Two representative H&E stained lung sections of the animals on the glucose-lowering regimen and B. control diet and no metformin. C. Scored metastatic burden in the lungs.

C

Since micrometastases are also characterized by hypoxia, we investigated whether metastasis was delayed in the PyMT transgenic mouse TNBC model treated with glucose-lowering therapy in comparison with control animals fed normal mouse chow without metformin. We examined the lungs *post-mortem* for the presence and size of metastases. Lungs were removed from euthanized mice, gently inflated and fixed in 10% NBF, embedded in paraffin. Paraffin-embedded lung tissue sections at least 300 µm apart were stained with H&E. Pathological examination was performed using 3 to 12 sections per animal and 3 animals from each group scored for metastatic burden (the ratio of metastatic area to the total lung area). As animals were terminated at different times, the metastatic burden to the lungs was age-adjusted according to the survival time from birth to the endpoint. Results were then averaged for each group. Control group - 1.7±0.6%, ketogenic diet plus metformin group – 0.5±0.2% (Representative images and metastatic burden are in Suppl. Fig. 2).

**Precision of tumor measurements with calipers**

Caliper measurement of small tumors can be imprecise. To account for measurement errors, three random independent measurements of 11 tumors in two dimensions were compared and relative deviations (sample standard deviation/mean) were calculated from their respective sample means. Elements of this vector *d_i_*, *i=*1, …66 come from the normal distribution (Lilliefors test, p>0.5). Then the precision of measurement is $PM=\sqrt{\frac{1}{n}\sum_{i=1}^{n} d_{i}^{2}}$= 0.0644 (or 6.4%), indicating the lower threshold for significant differences in tumor dimensions.
